# Model and Property Analysis for a Ball-Hinged Three-Degree-of-Freedom Piezoelectric Spherical Motor

**DOI:** 10.3390/s24051470

**Published:** 2024-02-24

**Authors:** Zhenyu Wang, Jun Li, Wanbing Liu, Guanshuai Jia, Ban Wang

**Affiliations:** 1 School of Mechanical Engineering, Hangzhou Dianzi University Information Engineering College, Hangzhou 311305, China; wzy19810911@163.com; 2 School of Mechanical Engineering, Hangzhou Dianzi University, Hangzhou 310018, China; 222010043@hdu.edu.cn; 3 Hengda Fuji Elevator Co., Ltd., Huzhou 313009, China; 4 College of Electrical Engineering, Zhejiang University, Hangzhou 310027, China; jiaguanshuai@163.com

**Keywords:** ball-hinged, three-degree-of-freedom, piezoelectric spherical motor, mathematical model, mechanical properties

## Abstract

Multi-degree-of-freedom piezoelectric motors have the advantages of high torque and resolution, simple structure, and direct drive, which are widely used in robot wrist joints, deep-sea mechanisms, medical equipment, and space mechanisms. To solve the problems of high force/torque coupling degree and ball low stator and rotor bonding strength of the traditional traveling wave type three-degree-of-freedom piezoelectric spherical motor, a new structure of ball-hinged piezoelectric spherical motor is proposed. Through coordinate transformation and force analysis, the driven mathematical model of the spherical motor is given. The model shows that the three degrees of freedom of the motor are coupled with each other. According to the mathematical model of the spherical motor, the mechanical properties of the motor are analyzed by the computer simulation. The results show that the stalling torque coefficient *k_t_* has a linear relationship with the friction coefficient *ε* and the stator preload *F*_c_, has a nonlinear relationship with the stator radius *R* and the rotor radius *r*, and increases with the increase of *R* and decreases with the increase of *r*. The no-load speed of motor ***ω_n_*** is not related to the friction coefficient *ε* and the stator preload *F*_c_, and increases with the increase of *R* and decreases with the increase of *r*. The anisotropic characteristics of torque and speed of a spherical motor are further analyzed, which lays a theoretical foundation for the drive control of a spherical motor.

## 1. Introduction

Multi-degree-of-freedom motors have always been the tireless pursuit of researchers in the field of electromechanical drives. With the development of the times, many new application scenarios have strongly demanded multi-degree-of-freedom motors, such as robot wrist joints, deep-sea mechanisms, space mechanisms, optical mechanisms, biomedical engineering, various types of pipelines, pumps and valves, gyroscopes, 3D spatial measurements, and many other occasions [1,2,3,4,5]. The initial multi-degree-of-freedom motor continued to use the principle of the electromagnetic motor, which made it difficult to form a continuous and stable space magnetic field. The motor structure was complex, making it difficult to manufacture and control. Many researchers have developed new methods. For example, multi-degree-of-freedom piezoelectric motors driven by ultrasonic vibration have the advantages of high torque and resolution, simple structure, and direct drive. Compared with electromagnetic motors, they have been applied in a variety of applications. In recent years, research on multi-degree-of-freedom piezoelectric spherical motors has become active.

The rotor of the multi-degree-of-freedom piezoelectric spherical motor is a sphere, which can realize 2 to 3 rotational degrees of freedom. It can be divided into the columnar stator type, annular stator type, and traveling wave stator type according to the shape characteristics of the stator. The columnar stator type was first proposed by Takemura [6]. The annular stator adopts a laminated structure, the metal elastic body and the piezoelectric element are clamped together by the screw (Langevin structure), and the two second-order bending vibration modes and one first-order longitudinal vibration mode of the vibrator are excited by alternating current in the ultrasonic frequency domain. The combination of three modes of vibration can cause elliptical motion of the particle on the driving end face of the stator, and then the spherical rotor is driven by friction to realize the three-degree-of-freedom motion of the motor. Nakamura et al. adopted two sets of piezoelectric vibrators to realize the short-column type stator structure, which was applied to the robot knuckle [7]. Hu Minqiang et al. adopted a double-stator clamping mechanism, which is more convenient for the stator to exert preload [8]. The columnar stator motor is relatively large in size, difficult to preload, and has a short life expectancy because of the point contact between the stator and rotor. The key point of the stator structure design is the consistency of bending and longitudinal vibration frequencies. The annular stator type has an annular stator, and the spherical rotor is driven by the combination of different vibration modes of the annular stator to achieve multi-degree-of-freedom motion. Two symmetrical stators are used to clamp the spherical rotor in the middle, which not only increases the output torque but also leaves the clamping mechanism of the spherical rotor out [9]. The piezoelectric ceramic ring is attached to the surface of the stator, and different combinations of voltage are applied to excite the longitudinal, bending, and torsional vibration modes to drive the rotation of the spherical rotor. The difficulty lies in the tuning of the three vibration modes because there is only one frequency for the driving power, and the motor lifespan is relatively short due to the point contact of the three modes. Moreover, the control strategy is complex, making it difficult for engineering applications.

The traveling wave stator type adopts three ring-shaped traveling wave stators to drive the spherical rotor. The three stators are arranged in space according to a certain pattern, which can achieve the three-degree-of-freedom rotation of the ball rotor. Toyama proposed the principle of this motor and carried out a series of studies [10,11,12]. Due to the reliable and stable ring traveling wave stator, which has a long lifespan and high force efficiency, this spherical motor has a higher efficiency and torque, approximately twice as large as that of a single traveling wave motor, although its speed will decrease slightly, making it more suitable for direct drive applications. By extending the driving concept of the traveling wave stator, a traveling wave copper wire ring can also be used as the driver, and its traveling wave is generated by a Langevin vibrator. Compared with the traditional traveling wave stator, this structure has a smaller volume, but the torque is relatively smaller [13,14]. Niu Zijie et al. proposed a three-stator piezoelectric spherical motor based on a flexible base, where the flexible base has an elastic structure to ensure a certain preload between the rotor and stator after assembly [15]. To adapt to the spherical rotor contact, Hu Xixing et al. proposed a stator structure with a large inclination angle at the outer edge and a line contact type at the inner edge, which significantly improved the energy exchange efficiency and stability of the stator [16]. In addition, a flexible plate spring is employed to adaptively compress the spherical rotor structure, making the motor structure simple, easy to assemble, and miniaturized. Due to the flexible plate spring compressed spherical rotor, the research result has shown that the position of the center of the spherical rotor is easily affected by external forces, which in turn affects the torque output. This situation is more severe for three-degree-of-freedom than for two-degree-of-freedom, so it is necessary to improve the motor structure to overcome this problem. Especially in some applications where motors are subjected to high forces, such as large spatial structures that are prone to large force/torque impacts, and axial instantaneous impact forces can reach thousands of Newtons, it is required that motors have good tensile/compressive capacity to avoid motor failure or even disassembly due to impact.

A ball-hinged piezoelectric spherical motor structure is proposed in this article, which solves the problem of poor tensile/compressive capacity of previous motors and difficulty in achieving precise control of spherical rotor movement. Based on previous work, a mathematical model for the motor drive is derived by coordinate transformation, and motor characteristics are analyzed under conditions such as stalling and no-load.

## 2. Structure of the Ball-Hinged Three-Degree-of-Freedom Piezoelectric Spherical Motor

**Figure 1:**
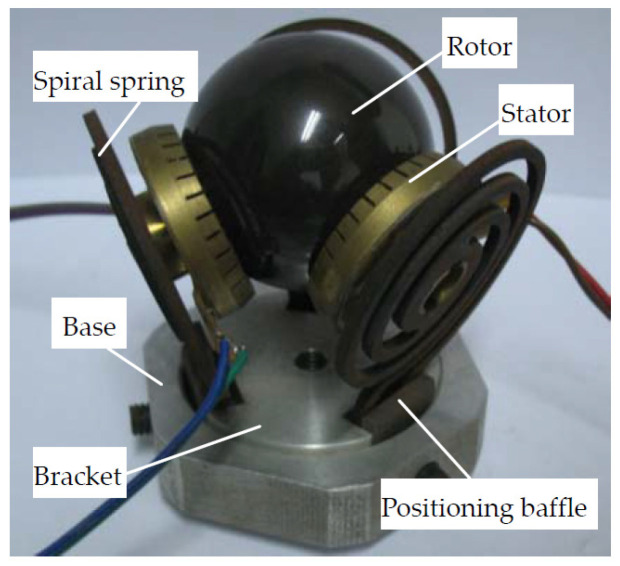
Structure of the traditional three-degree-of-freedom traveling wave motor.

The structure of the traditional three-degree-of-freedom traveling wave motor developed by our group in the early stages is shown in Figure 1. The rotor radius *R* is 20 mm, and the effective stator radius *r* is 12 mm. It mainly consists of components such as the base bracket, stator, rotor, positioning baffle, and spiral spring. The bracket is placed in the base, with a gap between them. The bracket is symmetrically distributed with three slots along the center of 120°, and three positioning baffles are placed in the slots, located in the gap between the spiral spring and the inner ring of the base. The bracket rises within the base when tightening the screw in the center of the base, causing the action point between the positioning plate and the spiral spring to descend relative to the spring. As a result, the three springs naturally expand outward, reducing the preload force between the traveling wave stator and the spherical rotor, and vice versa. This mechanism has the characteristics of a small size, a simple structure, and convenient installation and debugging.

**Figure 2:**
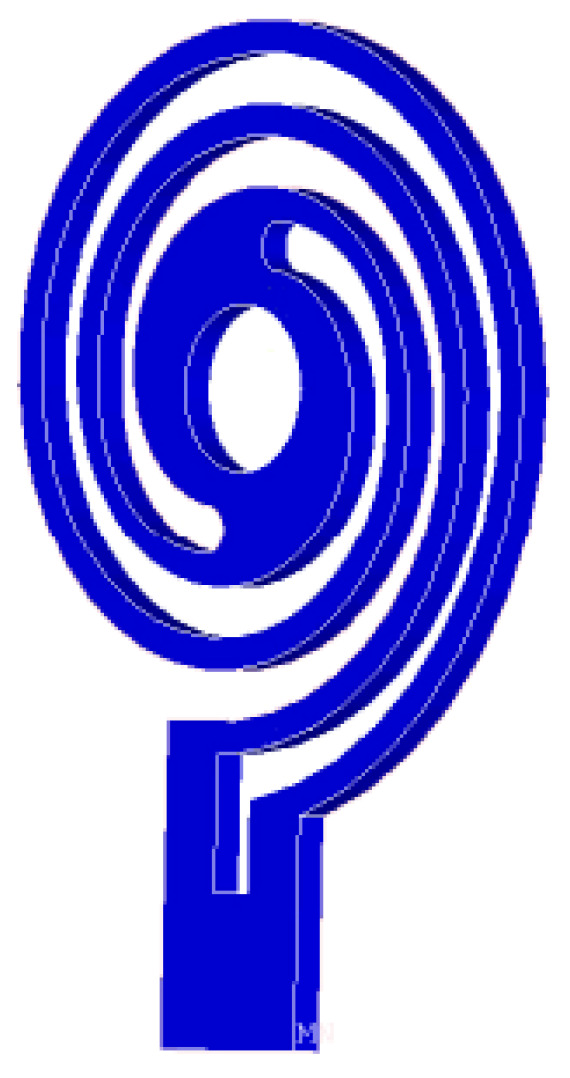
Spiral spring structure.

**Figure 3:**
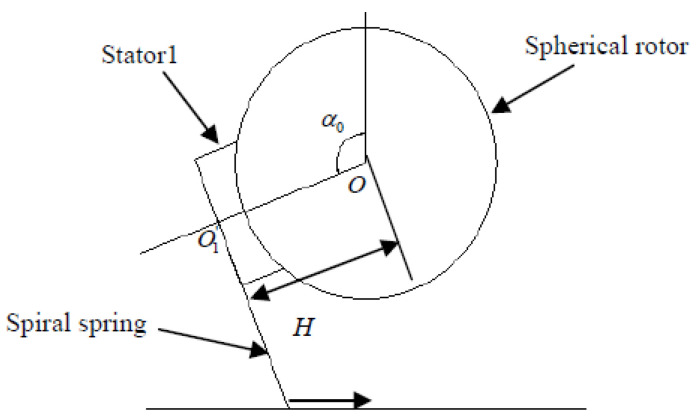
Spatial position diagram of components.

Flexible spiral plate springs (as shown in Figure 2) not only adaptively compress the spherical rotor but also automatically align the centers of the stator and rotor. However, in the case of an uneven load, the rotor center will move, which brings great difficulties to motor drive control. The detailed situation of the spiral spring motor is shown in Figure 3. When the preloading forces of the three spiral springs are different or the motor receives unbalanced external forces, the center position of the spherical rotor changes, which complicates the motor model and drive control. On the other hand, force transmission between the spherical rotor and stator is mainly achieved through flexible plate springs. The clamping force of the three stators holding the spherical rotor through the plate spring is too weak, without geometric rigidity constraints, and is easy to detach. In addition, the uneven force on the three stators under external force affects the output torque of the stator, which means that the motor torques under external force are coupled with each other.

**Figure 4:**
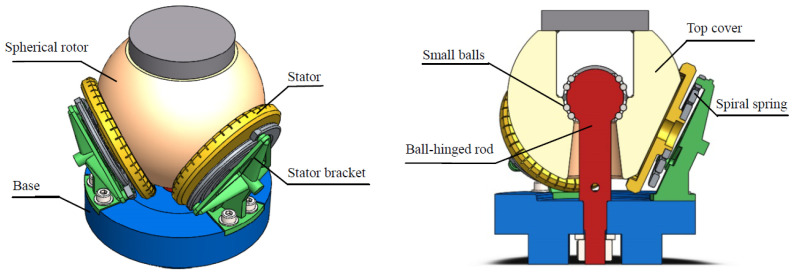
Structure of the ball-hinged, three-degree-of-freedom piezoelectric spherical motor.

A new structure of a multi-degree-of-freedom ball-hinged motor is proposed to solve the issues of rotor center movement, performance effects, and a complex mathematical model of the current piezoelectric spherical motor under external loads. As shown in Figure 4, a groove is set at the top of the spherical rotor, and an axial through-hole is set at the center of the bottom of the spherical rotor. The spherical structure of the ball-hinged rod is embedded in the hemispherical groove of the spherical rotor, and the top cover is fixed in the groove of the spherical rotor and covers the ball-hinged rod. The lower end of the ball-hinged rod passes down through the axial through-hole of the spherical rotor and is fixedly connected to the base. Small balls are installed at the rotating joint of the ball-hinged to reduce friction in the contact area. The pull/press forces of the motor act on the head of the ball-hinge rod, which greatly improves the strength of the motor against external forces. The built-in ball-hinged structure can fix the center of the spherical rotor, but the position of the center of the sphere can still be guaranteed in the case of unbalanced external forces. Due to the fixation of the center, the compression force between the spherical rotor and the stator remains constant, and the stator preload can be adjusted by changing the position of the stator bracket. The mathematical model of the ball-hinged motor drive is relatively simple, achieving the decoupling of force and torque for the spherical rotor. The spherical rotor has a small range of motion and is suitable for small displacement situations, such as vibration control of large spatial truss structures.

## 3. Driving Mathematical Model

**Figure 5:**
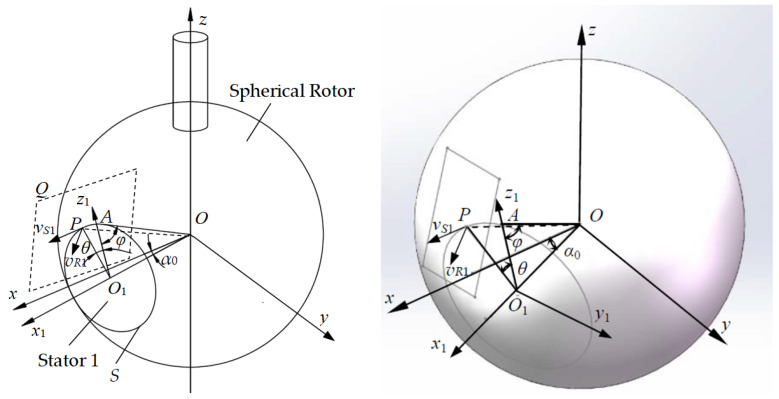
Model diagram of three-degree-of-freedom piezoelectric spherical motor.

The geometric relationship and spatial position relationship of the stator and rotor of the three-degree-of-freedom piezoelectric spherical motor are shown in Figure 5 [14]. Set the origin of the basic coordinate system Σ_0_(*Oxyz*) at point *O* of the spherical rotor center, where *α*_0_ represents the angle between the central axis of Stator 1 and the *x*-axis in the basic coordinate system Σ_0_, *S* represents the contact circumference of the stator and rotor, and the central axis of Stator 1 is located in the *x*-*z* plane. Let the radius of the spherical rotor be *R*, and the effective radius of the traveling wave Stator 1 be *r*, that is, the contact circumference radius between the stator and the spherical rotor is *r*. Set the upper intersection point between the contact circumference *S* of Stator 1 and the spherical rotor and the *x*-*z* plane as *A*. Let *O*_1_ be the center of the contact circumference *S*. The angle between *OA* and *O*_1_*A* is *φ*, and the angle between the line *O*_1_*P* (connecting *O*_1_ and any point *P* on the contact circumference *S*) and *O*_1_*A* is *θ*. The plane *Q* is perpendicular to the *OP* and passes through point *P*. The velocity vectors of the stator and rotor are distributed in the plane *Q*. The process of establishing the local coordinate system Σ_1_(*Ox*_1_*y*_1_*z*_1_) for Stator 1 is as follows: Rotate the basic coordinate system Σ_0_(Oxyz) around the *y*-axis by *α*_0_, align the *x*_1_-axis with the central axis of Stator 1, and keep *y*_1_ aligned with the *y*-axis. Rotate the local coordinate system Σ_1_(*Ox*_1_*y*_1_*z*_1_) of Stator 1 counterclockwise around the *z*-axis by 120° to obtain the local coordinate system Σ_2_(*Ox*_2_*y*_2_*z*_2_) for Stator 2. Rotate the local coordinate system Σ_1_(*Ox*_1_*y*_1_*z*_1_) of Stator 1 around the *z*-axis by 240° to obtain the local coordinate system Σ_3_(*Ox*_3_*y*_3_*z*_3_) for Stator 3.

**Figure 6:**
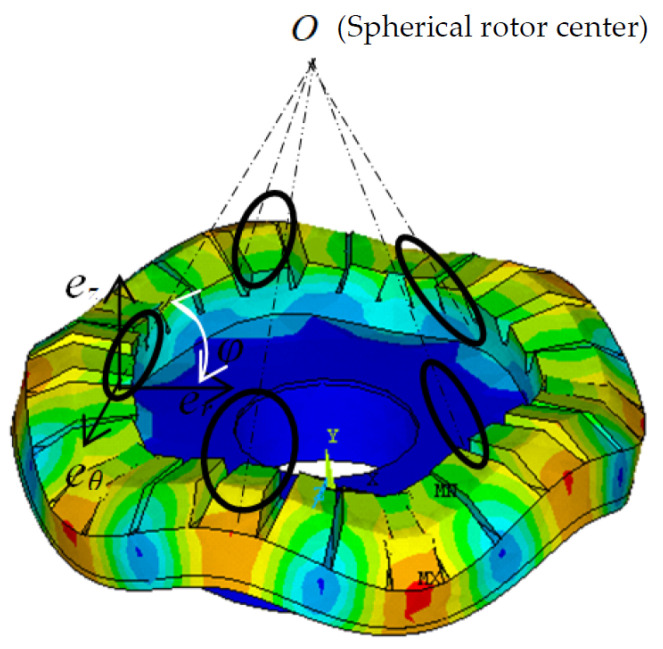
Motion diagram of stator surface particles.

We have studied a variety of multi-degree-of-freedom spherical motors in the early stages. The optimization design and analysis have been conducted on the stator of the spherical motor in preliminary research. The designed traveling wave stator is used to drive the spherical rotor, and the major axis of the oblique ellipse formed by the particles on the surface of the stator should point to the spherical rotor center *O,* as shown in Figure 6. The purpose of this design is to use the displacement component in the radial direction to increase the vibration amplitude on the contact interface between the stator and rotor, and improve the energy transfer efficiency of the motor.

Stator 1 is taken as an example for analysis, combined with the stator surface motion law shown in Figure 4. The angular velocity of the surface particles of traveling wave Stator 1 at the highest point of the elliptical motion track is *ω*_s1_, and the angular velocity of the ball rotor in the stator local coordinate system Σ_1_ is ***ω***_1_ = [*ω_x_*_1_ *ω_y_*_1_ *ω_z_*_1_]. The velocity ***υ***_S1_ at the highest point of the elliptical trajectory of the particle motion on the surface of the traveling wave Stator 1 is

(1) $$\begin{array}{cc} \upsilon_{S1} & =\omega_{s1}\times P_{\theta }\\ & =\omega_{s1}R{\left[\begin{array}{ccc} 0 & -\cos\varphi \cos\theta & -\cos\varphi \sin\theta \end{array}\right]}^{T} \end{array}$$

where

(2) $$P_{\theta }=R{\left[\begin{array}{ccc} \sin\varphi & -\cos\varphi \sin\theta & \cos\varphi \cos\theta \end{array}\right]}^{T}$$

is the vector from the spherical center to the contact point *P* of traveling wave stator 1, with the direction facing outward from the spherical center. The speed of the spherical rotor at contact point *P* of the stator and rotor ***υ****_R_*_1_ is

(3) $$\begin{array}{cc} \upsilon_{R1} & =\omega_{1}\times P_{\theta }\\ & =R\left[\begin{array}{l} \omega_{y1}\cos\varphi \cos\theta +\omega_{z1}\cos\varphi \sin\theta \\ \omega_{z1}\sin\varphi -\omega_{x1}\cos\varphi \cos\theta \\ -\omega_{y1}\sin\varphi -\omega_{x1}\cos\varphi \sin\theta \end{array}\right] \end{array}$$

As shown in Figure 3, the speed of the spherical rotor ***υ****_R_*_1_ is in the tangent plane of the contact point. For the convenience of analysis, ***υ****_R_*_1_ is decomposed into two parts: one is related to the ***υ***_S1_ direction consistent sub velocity ***υ****_y_*_1_, and the other part is the component velocity ***υ***_S1_ perpendicular to the ***υ****_x_*_1_ direction, with

(4) $$\begin{array}{cc} \upsilon_{y1} & =(\upsilon_{R1}\cdot t_{p})t_{p}\\ & =R(\omega_{x1}\cos\varphi +\omega_{y1}\sin\varphi \sin\theta \\ & \;-\omega_{z1}\sin\varphi \cos\theta )\left[\begin{array}{l} 0\\ -\cos\theta \\ -\sin\theta \end{array}\right] \end{array}$$

where ***t***_p_ = [0 −cos*θ* −sin*θ*] is the unit vector in the direction of ***υ****_y_*_1_.

(5) $$\begin{array}{cc} \upsilon_{x1} & =(\upsilon_{R1}\cdot n_{p})n_{p}\\ & =R(\omega_{y1}\cos\theta +\omega_{z1}\sin\theta )\left[\begin{array}{l} \cos\varphi \\ \sin\varphi \sin\theta \\ -\sin\varphi \cos\theta \end{array}\right] \end{array}$$

In Equation (5), ***n***_p_ is the unit vector in the direction of ***υ****_x_*_1_, which is

(6) $$n_{p}=\frac{P_{\theta }}{|P_{\theta }|}\times t_{p}=\left[\begin{array}{l} \cos\varphi \\ \sin\varphi \sin\theta \\ -\sin\varphi \cos\theta \end{array}\right]$$

Using the classical friction model [17], the driving force in the ***υ***_S1_ direction generated by traveling wave Stator 1 on the spherical rotor is

(7) $$\begin{array}{cc} F_{d1} & =\varepsilon (\upsilon_{S1}-\upsilon_{y1})F_{n1}\\ & =\varepsilon F_{n1}R[(\omega_{x1}\cos\varphi +\omega_{y1}\sin\varphi \sin\theta )\\ & \;-\omega_{z1}\sin\varphi \cos\theta -\omega_{s1}\cos\varphi ]\left[\begin{array}{c} 0\\ \cos\theta \\ \sin\theta \end{array}\right] \end{array}$$

where ε is the coefficient of friction and the driving torque is

(8) $$\begin{array}{cc} T_{d1} & =\frac{n}{2\pi }\int_{0}^{2\pi }P_{\theta 1}\times F_{d1}d\theta \\ & =\frac{n\varepsilon F_{n1}R^{2}}{2}\left[\begin{array}{c} 2(\omega_{s1}-\omega_{x1})\cos^{2}\varphi \\ -\omega_{y1}\sin^{2}\varphi \\ -\omega_{z1}\sin^{2}\varphi \end{array}\right] \end{array}$$

The resistance expression is as follows:

(9) $$\begin{array}{cc} F_{f1} & =\varepsilon (0-\upsilon_{x1})F_{n1}\\ & =\varepsilon F_{n1}R(\omega_{y1}\cos\theta +\omega_{z1}\sin\theta )\left[\begin{array}{c} -\cos\varphi \\ -\sin\varphi \sin\theta \\ \sin\varphi \cos\theta \end{array}\right] \end{array}$$

and the resistance torque is

(10) $$T_{f1}=\frac{n}{2\pi }\int_{0}^{2\pi }P_{\theta 1}\times F_{f1}d\theta =-\frac{n\varepsilon F_{n1}R^{2}}{2}\left[\begin{array}{c} 0\\ \omega_{y1}\\ \omega_{z1} \end{array}\right]$$

The total torque generated by Stator 1 on the spherical rotor is

(11) $$\begin{array}{cc} T_{1} & =T_{d1}+T_{f1}\\ & =\frac{n}{2}\varepsilon F_{n1}R^{2}\left[\begin{array}{c} 2\omega_{s1}\cos^{2}\varphi -2\omega_{x1}\cos^{2}\varphi \\ -\omega_{y1}\sin^{2}\varphi -\omega_{y1}\\ -\omega_{z1}\sin^{2}\varphi -\omega_{z1} \end{array}\right] \end{array}$$

Let

(12) $$\omega_{s1}={\left[2\omega_{s1}\cos^{2}\varphi \;0\;0\right]}^{T}$$

(13) $$K=\left[\begin{array}{ccc} -2\cos^{2}\varphi & 0 & 0\\ 0 & -(1+\sin^{2}\varphi ) & 0\\ 0 & 0 & -(1+\sin^{2}\varphi ) \end{array}\right]$$

(14) $$\omega_{1}={\left[\omega_{x1}\;\omega_{y1}\;\omega_{z1}\right]}^{T}$$

Equation (11) can be written in the following matrix form:

(15) $$T_{1}=\omega_{s1}+K\omega_{1}$$

Similarly, the total torque of Stator 2 on the spherical rotor in the local coordinate system Σ_2_(*Ox*_2_*y*_2_*z*_2_) and the total torque of Stator 3 on the ball rotor in the local coordinate system Σ_3_(*Ox*_3_*y*_3_*z*_3_) are

(16) $$T_{2}=\omega_{s2}+K\omega_{2}$$

(17) $$T_{3}=\omega_{s3}+K\omega_{3}$$

where

(18) $$\omega_{s2}=[2\omega_{s2}\cos^{2}\varphi \;0\;0]$$

(19) $$\omega_{2}={\left[\omega_{x2}\;\omega_{y2}\;\omega_{z2}\right]}^{T}$$

(20) $$\omega_{s2}=[2\omega_{s3}\cos^{2}\varphi \;0\;0]$$

(21) $$\omega_{3}={\left[\omega_{x3}\;\omega_{y3}\;\omega_{z3}\right]}^{T}$$

Thus far, the total torque of the three stators in their respective local coordinate systems has been obtained. To facilitate the calculation of the total torque of the three stators to the ball rotor, it is necessary to equivalently transform the torque and speed into the basic coordinate system Σ_0_(*Oxyz*) through coordinate transformation.

According to the coordinate rotation transformation relationship, the rotation transformation matrix from the Stator 1 local coordinate system Σ_1_ to the basic coordinate system Σ_0_ is

(22) $$A_{10}=\left[\begin{array}{ccc} \cos\alpha_{0} & 0 & -\sin\alpha_{0}\\ 0 & 1 & 0\\ \sin\alpha_{0} & 0 & \cos\alpha_{0} \end{array}\right]$$

The rotation transformation matrix from the basic coordinate system Σ_0_ to the Stator 1 local coordinate system Σ_1_ is

(23) $$A_{01}=\left[\begin{array}{ccc} \cos\alpha_{0} & 0 & \sin\alpha_{0}\\ 0 & 1 & 0\\ -\sin\alpha_{0} & 0 & \cos\alpha_{0} \end{array}\right]$$

The rotation transformation matrix from the Stator 2 local coordinate system Σ_2_ to the basic coordinate system Σ_0_ is

(24) $$A_{20}=\left[\begin{array}{ccc} -1/2 & \sqrt{3}/2 & 0\\ -\sqrt{3}/2 & -1/2 & 0\\ 0 & 0 & 1 \end{array}\right]\left[\begin{array}{ccc} \cos\alpha_{0} & 0 & -\sin\alpha_{0}\\ 0 & 1 & 0\\ \sin\alpha_{0} & 0 & \cos\alpha_{0} \end{array}\right]$$

The rotation transformation matrix from the basic coordinate system Σ_0_ to the Stator 2 local coordinate system Σ_2_ is

(25) $$A_{02}=\left[\begin{array}{ccc} \cos\alpha_{0} & 0 & \sin\alpha_{0}\\ 0 & 1 & 0\\ -\sin\alpha_{0} & 0 & \cos\alpha_{0} \end{array}\right]\left[\begin{array}{ccc} -1/2 & -\sqrt{3}/2 & 0\\ \sqrt{3}/2 & -1/2 & 0\\ 0 & 0 & 1 \end{array}\right]$$

The rotation transformation matrix from the Stator 3 local coordinate system Σ_3_ to the basic coordinate system Σ_0_ is

(26) $$A_{30}=\left[\begin{array}{ccc} -1/2 & -\sqrt{3}/2 & 0\\ \sqrt{3}/2 & -1/2 & 0\\ 0 & 0 & 1 \end{array}\right]\left[\begin{array}{ccc} \cos\alpha_{0} & 0 & -\sin\alpha_{0}\\ 0 & 1 & 0\\ \sin\alpha_{0} & 0 & \cos\alpha_{0} \end{array}\right]$$

The rotation transformation matrix from the basic coordinate system Σ_0_ to the Stator 3 local coordinate system Σ_3_ is

(27) $$A_{03}=\left[\begin{array}{ccc} \cos\alpha_{0} & 0 & \sin\alpha_{0}\\ 0 & 1 & 0\\ -\sin\alpha_{0} & 0 & \cos\alpha_{0} \end{array}\right]\left[\begin{array}{ccc} -1/2 & \sqrt{3}/2 & 0\\ -\sqrt{3}/2 & -1/2 & 0\\ 0 & 0 & 1 \end{array}\right]$$

According to the coordinate transformation, for Stator 1

(28) $$\left\{\begin{array}{l} T_{10}=A_{10}T_{1}\\ \omega_{1}=A_{01}\omega_{0} \end{array}\right.$$

The total torque generated by Stator 1 on the spherical rotor in the basic coordinate system Σ_0_ is

(29) $$T_{10}=A_{10}(\omega_{s1}+KA_{01}\omega_{0})$$

Similarly,

(30) $$T_{20}=A_{20}(\omega_{s2}+KA_{02}\omega_{0})$$

(31) $$T_{30}=A_{30}(\omega_{s3}+KA_{03}\omega_{0})$$

On the other hand, in the stator local coordinate system, the force balance formula can be obtained.

(32) $$\frac{n}{2\pi }\int_{0}^{2\pi }\left(F_{\mathrm{ni}}P_{e}-F_{\mathrm{di}}-F_{\mathrm{fi}}\right)\cdot x_{1}d\theta =F_{\mathrm{ci}}$$

where

(33) $$P_{e}={\left[\begin{array}{ccc} \sin\varphi & -\cos\varphi \sin\theta & \cos\varphi \cos\theta \end{array}\right]}^{T}$$

is the unit vector from *O* to point *P*, ***x****_i_* is the unit vector from the spherical center *O* to the center of the *i*-th traveling wave stator, with the direction of the spherical center outwards, and *F*_ci_ is the preload applied to the traveling wave stator. According to the above equation, the following can be concluded:

(34) $$F_{\mathrm{ni}}=\frac{1}{n\sin\varphi }F_{ci}$$

For the balance of motor force and the convenience of control, generally the three stators of the motor are loaded with the same preload, that is,

(35) $$F_{c1}=F_{c2}=F_{c3}$$

The matrix form is

(36) $$T_{0}=k(C\omega_{s}+D\omega_{0})$$

In the equation

(37) $$k=\frac{\varepsilon F_{c}R^{2}}{2\sin\varphi }$$

(38) $$C=\cos^{2}\varphi \left[\begin{array}{ccc} 2\cos\alpha_{0} & -\cos\alpha_{0} & -\cos\alpha_{0}\\ 0 & -\sqrt{3}\cos\alpha_{0} & \sqrt{3}\cos\alpha_{0}\\ 2\sin\alpha_{0} & 2\sin\alpha_{0} & 2\sin\alpha_{0} \end{array}\right]$$

(39) $$D=\left[\begin{array}{lll} d_{11} & d_{12} & d_{13}\\ d_{21} & d_{22} & d_{33}\\ d_{31} & d_{32} & d_{33} \end{array}\right]$$

where

(40) $$\left\{\begin{array}{cc} d_{11}= & -\frac{1}{2}\sin^{2}\alpha_{0}{(1+\sin}^{2}\varphi )-\frac{3}{2}\sin^{2}\varphi \\ & -\cos^{2}\varphi (2\cos\alpha_{0}+\cos^{2}\alpha_{0})-\frac{3}{2}\\ d_{12}= & 0\\ d_{13}= & 2\sin\alpha_{0}\cos\alpha_{0}\cos^{2}\varphi +\sin\alpha_{0}{(1+\sin}^{2}\varphi )\\ & -\sin\alpha_{0}\cos\alpha_{0}{(1+\sin}^{2}\varphi )\\ d_{21}= & 0\\ d_{22}= & \left(\frac{3}{2}\sin^{2}\alpha_{0}{(1+\sin}^{2}\varphi )-\frac{3}{2}(1+\sin^{2}\varphi \right)\\ & -3\cos^{2}\alpha_{0}\cos^{2}\varphi )\\ d_{23}= & 0\\ d_{31}= & 2\sin\alpha_{0}\cos\alpha_{0}\cos^{2}\varphi -2\sin\alpha_{0}\cos^{2}\varphi \\ & -\sin\alpha_{0}\cos\alpha_{0}{(1+\sin}^{2}\varphi )\\ d_{32}= & 0\\ d_{33}= & -2\cos^{2}\alpha_{0}{(1+\sin}^{2}\varphi )-\cos\alpha_{0}{(1+\sin}^{2}\varphi )\\ & -4\sin^{2}\alpha_{0}\cos^{2}\varphi \end{array}\right.$$

## 4. Mechanical Properties

It is known that the motor output torque can be controlled by the circumferential angular velocity ***ω***_s_ of the peak point of the traveling wave on the surface of the motor stator from Equation (36). The three rotation degrees of freedom of the spherical motor are coupled with each other, so the three stators need to cooperate with each other to realize the multi-degree-of-freedom motion and control of the motor.

### 4.1. Locked Characteristics

When the motor rotor is locked, we obtain

(41) $$T_{st}=kC\omega_{s}=\frac{\varepsilon F_{c}R^{2}\cos^{2}\varphi }{2\sin\varphi }\left[\begin{array}{l} \cos\alpha_{0}(2\omega_{s1}-\omega_{s2}-\omega_{s3})\\ \sqrt{3}\cos\alpha_{0}(-\omega_{s2}+\omega_{s3})\\ 2\sin\alpha_{0}(\omega_{s1}+\omega_{s2}+\omega_{s3}) \end{array}\right]$$

We define the following new parameter as

(42) $$k_{t}=\frac{\varepsilon F_{c}R^{2}\cos^{2}\varphi }{2\sin\varphi }=\frac{\varepsilon RF_{c}r^{2}}{2\sqrt{R^{2}-r^{2}}}$$

The locked torque constant *k_t_* reflects the strength of the starting ability of the motor. It can be seen from its expression that it has a linear relationship with the friction coefficient *ε* and the stator preload *F_c_*, and it has a nonlinear relationship with the stator radius *R* and *r*.

**Figure 7:**
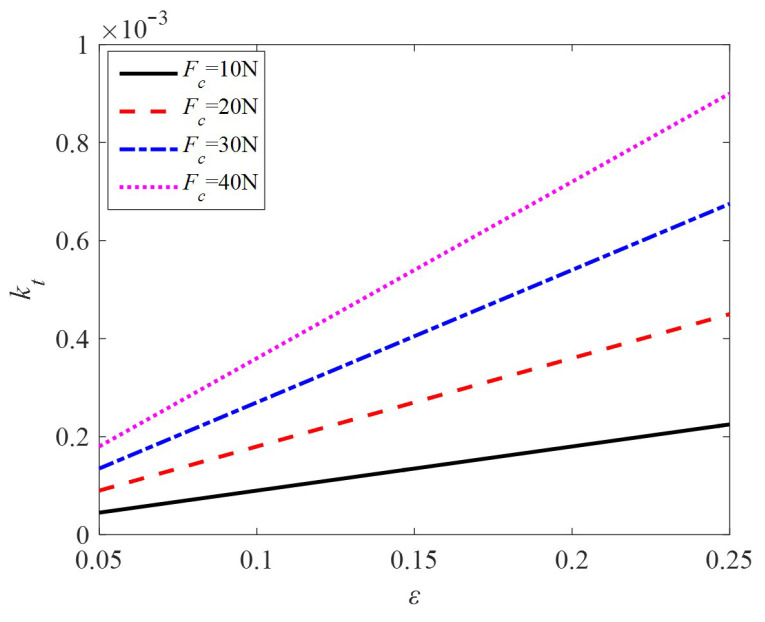
The relationship curve between *k_t_* and *ε.*

Taking the relevant motor parameters *R* = 20 mm, *r* = 12 mm, and modal order *n* = 5, the relationship between the locked torque constant *k_t_* and the friction coefficient *ε* under different preloads is shown in Figure 7. It is obvious that the larger the friction coefficient is, the larger the torque constant will be.

**Figure 8:**
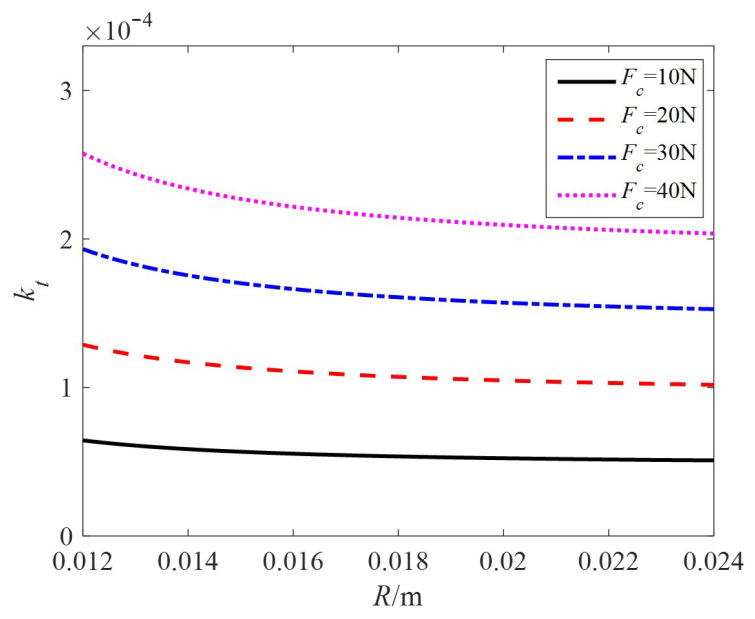
The relationship curve between *k_t_* and *R.*

Taking the relevant motor parameters *ε* = 0.15, *r* = 8 mm, and modal order *n* = 5, the relationship between the locked torque constant kt and the stator radius *R* under different preloads is shown in Figure 8. It can be seen that the larger the radius, the smaller the locked torque constant under the same rotor radius, and the larger the preload, the larger the torque constant.

**Figure 9:**
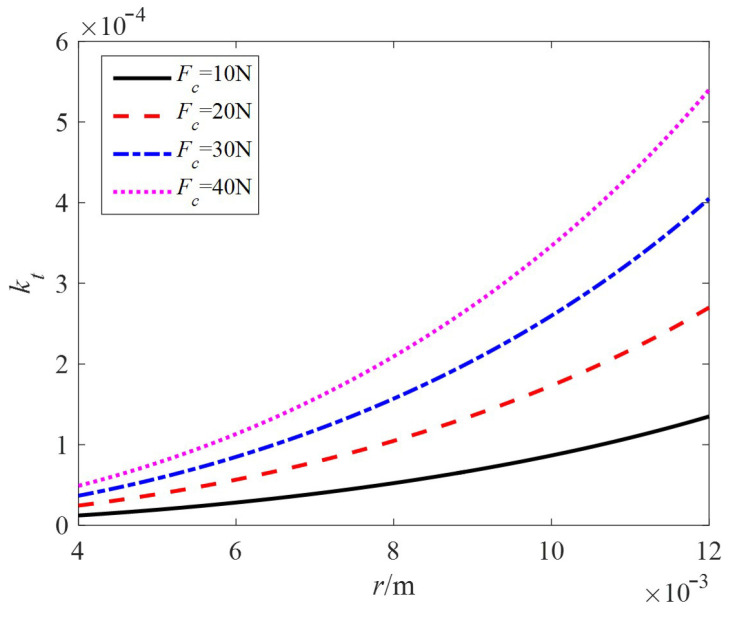
The relationship curve between *k_t_* and *r.*

Taking the relevant motor parameters *ε* = 0.15, *R* = 20 mm, and modal order *n* = 5, the relationship between the locked torque constant and stator radius *r* under different preloads is shown in Figure 9. The larger the radius is, the larger the locked torque constant; the larger the preload is, the larger the torque constant under the same stator radius.

### 4.2. No-Load Characteristics

Let the motor torque ***T***_0_ = 0, and the no-load speed of the motor can be obtained as

(43) $$\omega_{n}=-D^{-1}C\omega_{s}$$

Bringing matrices ***D*** and ***C*** into Equation (43), it is shown that the no-load speed ***ω****_s_* has nothing to do with the friction coefficient *ε* and the stator preload *F_c_*, but it is related to the stator tilt angle *α*_0_, the rotor radius *R*, and the stator diameter *r*. The stator inclination angle is generally determined by the structure of the motor. The influence of the radius of the stator and rotor on the no-load speed is analyzed as follows.

**Figure 10:**
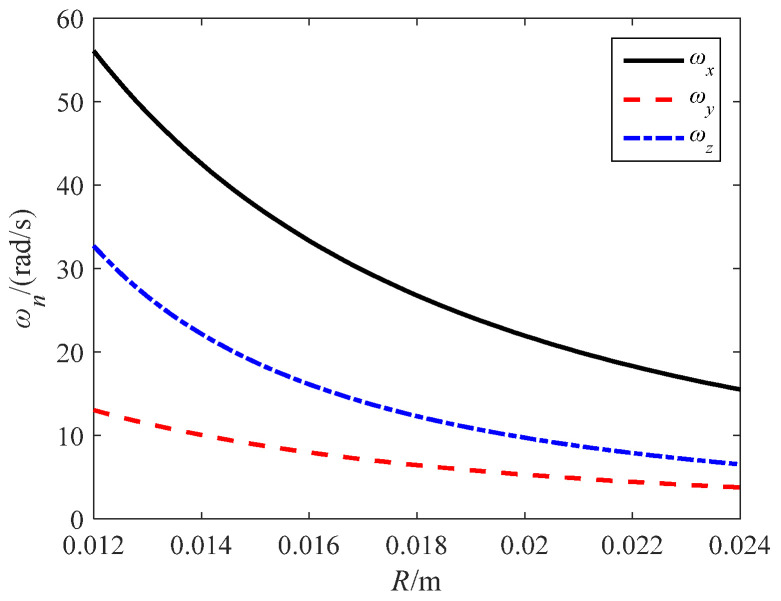
The relationship curve between *ω_n_* and *R.*

During the simulation process, the no-load speed is guaranteed to be greater than 0. Take *ω_s_*_1_ = 400 rad/s, *ω_s_*_2_ = 140 rad/s, and *ω_s_*_3_ = 200 rad/s. Taking *r* = 8 mm and *α*_0_ = 15°, the relationship between the speed components of the no-load speed and the rotor radius *R* is shown in Figure 10. As the rotor radius *R* increases, the components of the no-load speed gradually decrease.

**Figure 11:**
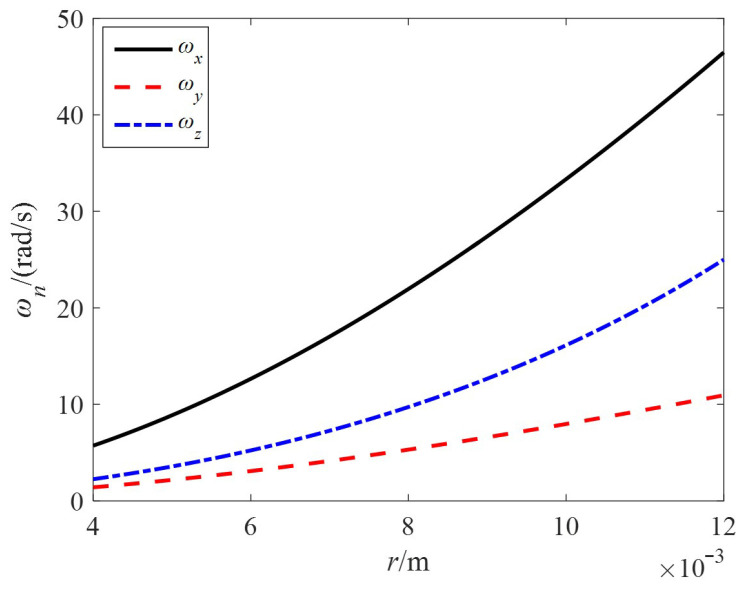
The relationship curve between ***ω_n_*** and *r.*

Taking *R* = 20 cm and *α*_0_ = 15°, the relationship between the speed components of the no-load speed and the stator radius *r* is shown in Figure 11. As the stator radius *r* increases, the components of the no-load speed gradually increase.

### 4.3. Torque and Speed Anisotropy

**Figure 12:**
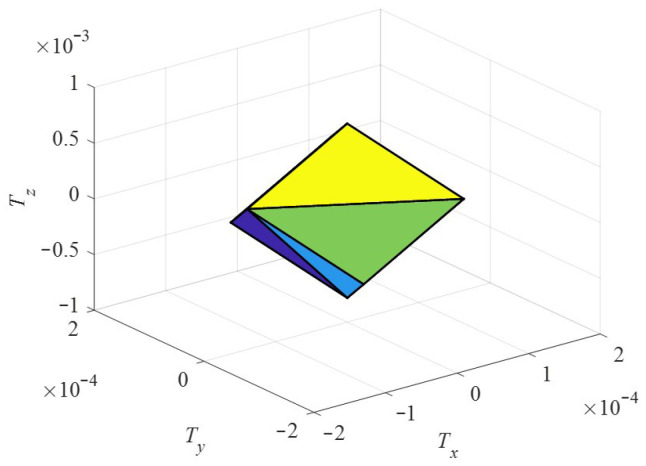
Distribution diagram of the locked torque.

Assuming that the maximum angular velocity of the three stators is within the unit range, that is, *ω*_s1_, *ω*_s2_, *ω*_s3_ ∈ [−1,1] the relationship between the modulus of the total torque *T*_st_ of the motor stall and the component torque can be obtained. Taking motor related parameters *ε* = 0.15, *r* = 12 mm, *R* = 20 mm, modal order *n* = 5 and preload force *F_c_* = 10 N, the distribution of the locked torque of the motor in the torque space is shown in Figure 12. The torque space is distributed as a hexahedron, and the maximum value of the locked torque *T*_st_ of the motor in different directions is different, showing torque anisotropy.

**Figure 13:**
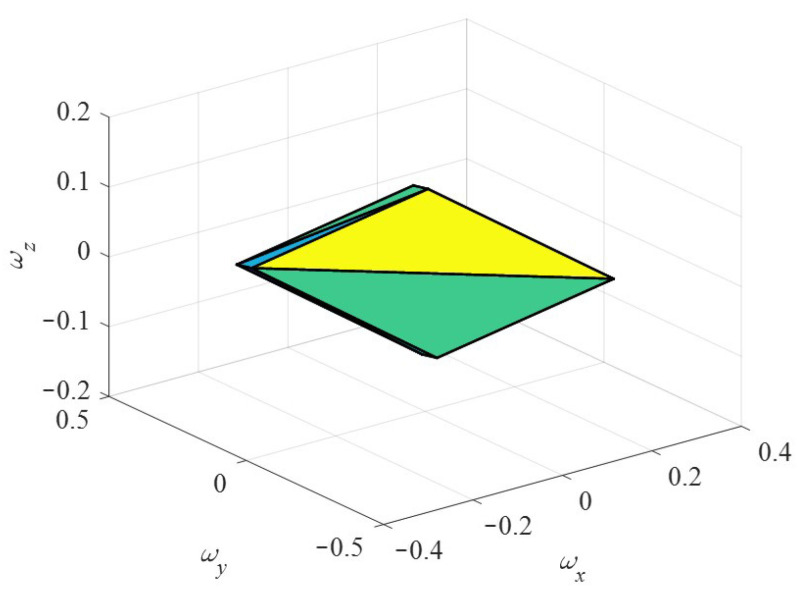
Distribution diagram of the no-load speed.

In the same way, the relationship between the modulus value of the no-load speed ***ω_n_*** of the motor and the speed component can be obtained. The distribution of the no-load speed ***ω_n_*** of the motor in the speed space is shown in Figure 13. The speed space distribution is also a hexahedron, and its shape is similar to the spatial distribution diagram of the locked torque. The maximum value of the resultant speed of the motor in different directions is different, showing speed anisotropy.

## 5. Conclusions

This article proposes a ball-hinged, three-degree-of-freedom piezoelectric spherical motor structure that solves the problem of force/torque coupling in traditional flexible plate spring structures. On the basis of the previous research on the stator design, the driving mathematical model of the spherical motor is given, which provides a model basis for the driving control of the motor. According to the mathematical model of the motor, the influence of various parameters of the motor on the mechanical characteristics of the motor is simulated, and the conclusions are as follows.

(1) The proposed new structure of the motor can fix the center of the spherical rotor, which has the advantages of a simple driving model, force/moment decoupling, and high structural strength.

(2) The parameters such as stator radius *r*, rotor radius *R*, preload *F_c,_* and friction coefficient *ε* have a great impact on the motor mechanical properties, and different motor parameters can be selected according to needs.

(3) Because the stators are placed in the middle and lower parts of the rotor, the locked torque and no-load speed are not the same in different directions. That is, they have anisotropy, which needs to be paid attention to when doing motor applications in the future.

The work in this manuscript provides a theoretical basis for the next step in the optimal design of the motor. In the future, the motor prototype and drive control strategy will be developed, and the practical application of spherical motors will be actively promoted in space truss vibration control, robot wrist joints, and multi-degree-of-freedom cameras.

## Data Availability

The data and materials in this article are made available at reasonable request.
